# Associations of pre-existing comorbidities and COVID-19 in-hospital mortality: an unCoVer analyses: Elly Mertens

**DOI:** 10.1093/eurpub/ckac130.015

**Published:** 2022-10-25

**Authors:** E Mertens, E Ademovic, M Majdan, JB Soriano, AC Trofor, JL Peñalvo

**Affiliations:** 1 Unit of Non-Communicable Diseases, Institute of Tropical Medicine, Antwerp, Belgium; 2 Epidemiology and Biosttatistics, University of Sarajevo, Sarajevo, Bosnia and Herzegovina; 3 Institute for Global Health and Epidemiology, Trnava University, Trnava, Slovakia; 4 Neumology Service, University Hospital La Princesa, Madrid, Spain; 5 CIBERES, Institute de Salud Carlos III, Madrid, Spain; 6 COVID-19 Clinical Management Team, WHO Health Emergency, Geneva, Switzerland; 7 Clinical Hospital of Pulmonary Diseases Iasi, Clinical Hospital of Pulmonary Diseases Iasi, Iasi, Romania; 8 University of Medicine and Pharmacy, Grigore T. Popa Iasi, Iasi, Romania

## Abstract

**Background:**

Accumulated evidence on risk factors for adverse COVID-19 outcomes revealed that old age and male sex are main associates, next to pre-existing comorbidities, as analysed from scattered single cohorts of hospitalised COVID-19 patients of accessible electronic medical records. Hence, evidence from federated analyses is called for to provide a more comprehensive and robust analyses of risk factors.

**Methods:**

Using the unCoVer network, i.e., a research platform of 29 partners for the expert use of patient data as routinely gathered in real-world healthcare settings, present analyses restricted to available data of four hospitals from Spain, Slovakia, Romania and Bosnia and Herzegovina covering 8,287 hospitalised COVID-19 patients. In-hospital death after COVID-19 diagnosis was examined in relation to common pre-existing comorbidities using virtual pooling of logistic regression models adjusted for age and sex.

**Results:**

Patients were on average 60.1 (± 20.9) years, 50.7% were male, almost half (43.3%) had at least one pre-existing comorbidity (17.4% having obesity, 21.9% hypertension, 18.0% diabetes and 13.7% cardiovascular diseases (CVD)), and 12.6% died during hospitalisation. Patients with comorbidities had a higher risk of mortality that was increasing with the number of comorbidities: from a virtual pooled odds ratio of 1.16 (95%CI: 0.96, 1.40) for one vs none to 1.30 (1.04, 1.64) and 2.14 (1.64, 2.79) for two and three or more comorbidities, respectively. Of the comorbidities, highest risk was seen for CVD (1.68; 1.40, 2.01), followed by hypertension (1.40; 1.19, 1.64) and diabetes (1.27; 1.07, 1.50), and the lowest for obesity (1.13; 0.94, 1.37).

**Conclusions:**

By federated analyses, this study confirmed that the number of comorbidities was a strong risk factor for in-hospital death after COVID-19, in particular CVD. The unCoVer platform pursues using scattered data sources by innovative computational resources and integrated information for enhanced impact.

**Key messages:**

- Federated analyses, capable of streamlining ethical and legal aspects, provide unique opportunities for robust results to inform public health.
- Higher COVID-19 in-hospital mortality risk with increasing number of comorbidities.

